# Immunoglobulin Rapid Test Sensitivity in PCR-Positive COVID-19 Patients

**DOI:** 10.1007/s44229-022-00014-x

**Published:** 2022-07-07

**Authors:** Ahmad A. Alharbi, Mohammad K. Alshomrani, Abdullah A. Alharbi, Abdulrahman H. Almaeen, Saad AlAsiri, Awad Al-Omari, Imad Alishat, Saeed Dolgom

**Affiliations:** 1 Department of Pathology, College of Medicine, Majmaah University and Dr. Sulaiman Al-Habib Hospital in AL Takhassusi, Riyadh, Kingdom of Saudi Arabia; 2 Microbiology Department, Riyadh Regional Laboratory and Blood Bank, Riyadh, Kingdom of Saudi Arabia; 3 Pathology Department, College of Medicine, Imam Muhammad Ibn Saud Islamic University, Riyadh, Kingdom of Saudi Arabia; 4 Department of Pathology, College of Medicine, Jouf University, Al-Jouf, Kingdom of Saudi Arabia; 5 Emergency Medicine, Dr. Sulaiman Al-Habib Medical Group, Riyadh, Kingdom of Saudi Arabia; 6 Dr. Sulaiman Al Habib Medical Group and Alfaisal University, Riyadh, Kingdom of Saudi Arabia; 7 Emergency Department, Takhassusi Hospital, Dr. Sulaiman AlHabib Medical Group, Riyadh, Kingdom of Saudi Arabia; 8 Pediatric Infectious Diseases, Dr.Suliman ALhabib Hospital, Riyadh, Kingdom of Saudi Arabia

**Keywords:** COVID-19, Rapid serological test, IgG, IgM, Sensitivity

## Abstract

**Background:**

Diagnostic assays aimed at the identification of immunoglobulin G (IgG) and immunoglobulin M (IgM) offer a rapid and adjunct modality to conventional real-time reverse transcription polymerase chain reaction (rRT-PCR) assays for the diagnosis of coronavirus disease 2019 (COVID-19).

**Aim:**

To analyze the sensitivity of IgG and IgM-based serological assays in rRT-PCR-positive COVID-19 subjects.

**Methods:**

A consecutive cohort of 69 patients with COVID-19-related symptoms or recent exposure to COVID-19-positive individuals were included after taking informed consent. Nasopharyngeal swabs for SARS-CoV-2 rRT-PCR analysis and venous blood samples for the COVID-19 IgG/IgM rapid test were simultaneously collected from each subject on day 0. Then, in the case of positive PCR results, subsequent blood samples for COVID-19 IgG/IgM analysis were collected on days 7, 10 and 14. Samples were statistically analyzed to determine the sensitivity of the serology-based assays.

**Results:**

No correlation was found between age or sex and the rRT-PCR, IgG and IgM results; 65.2% of subjects tested positive by rRT-PCR. The sensitivity of the IgM and IgG rapid test increased gradually with time, reaching the highest level on day 14 (22.2% and 72%, respectively).

**Conclusion:**

Serological assays for the detection of infection with SARS-CoV-2 were compared to rRT-PCR. These assays yielded lower sensitivities than rRT-PCR-based assays. However, given that these immunoassays are more affordable, faster, and easier to execute, they could be recommended for epidemiological research or characterizing the immune status of post-infection or post-vaccination subjects.

## Introduction

Coronavirus disease 2019 (COVID-19) is caused by severe acute respiratory syndrome coronavirus 2 (SARS-CoV-2) and has wreaked havoc on healthcare and economic systems worldwide. Despite extensive scientific research and efforts, there are still knowledge gaps regarding pathophysiology, clinical severity, and diagnosis in the COVID-19 patient population. There are various diagnostic approaches for SARS-CoV-2; however, the most commonly used techniques include the molecular detection of viral RNA, serological screening for antibodies and the detection of viral antigens [1]. Real-time reverse transcription-polymerase chain reaction (rRT-PCR) is the gold standard and the most sensitive test for diagnosing SARS-CoV-2 infection and detects RNA in upper and lower respiratory tract samples. Conventional PCR is laborious, time consuming and requires specialization in terms of instrumentation and the analysis of results. On the other hand, fully automated, new methods of PCR have emerged that are more user-friendly. However, higher costs and the inappropriateness of such PCR-based assays for follow up, where infected people may continue to have detectable RNA after recovery, represent non-negligible drawbacks [1, 2]. Antigen tests are immunoassays that detect a specific viral antigen that is indicative of current viral infection. The nasopharynx, nose or saliva are currently approved sites for performing antigenic testing. These tests are rapid and easy to use, generating results within 15–30 min. There are multiple authorized settings for the administration of antigen tests including point-of-care, laboratory-based and self-tests [3]. However, antigen tests for SARS-CoV-2 are generally less sensitive than rRT-PCR and other nucleic acid amplification tests (NAATs) [2].

Antibodies are markers for determining protection and immune status; however, some patients with antibodies are amenable for reinfection. Antibody detection is useful for affirming prior exposure to SARS-CoV-2 that may be missed by RT-PCR. However, this method should not be used solely to diagnose current infection because it may take a few weeks after infection to become positive [4].

Serological tests for COVID-19 are based on the detection of immunoglobulin (Ig) G and IgM antibodies against SARS-CoV-2 antigens. Various assays have been developed to detect these antibodies in human samples, including enzyme-linked immunosorbent assays (ELISAs), chemiluminescence enzyme immunoassays (CLIAs), fluorescence immunoassays (FIAs), and the lateral flow immunoassays (LFIAs). Based on technical and methodological aspects, these assays appear to differ in terms of their overall performance, sensitivity, specificity, and ability to measure IgM and IgG separately or in combination. With regard to the serological assays, the pooled sensitivity of ELISAs measuring IgG or IgM was determined to be 84.3% (95% confidence interval, CI, 75.6–90.9%); that of LFIAs was 66% (95% CI 49.3–79.3%) and that of CLIAs was 97.8% (95% CI 46.2–100%) [5].

The genome of COVID-19 is 30 kilobases in size and encodes structural proteins including the membrane (M), envelope (E), nucleocapsid (N) and spike (S) proteins. The binding of the S protein domain with the host cell surface receptor, i.e., angiotensin-converting enzyme 2 (ACE2), leads to viral infection, immune system activation and antibody production [6]. Antibodies against SARS-CoV-2 start to appear in COVID-19 patients 3–5 days after infection with the virus and increase over time; therefore, the sensitivity of the serological tests for SARS-CoV-2 gradually increases post-infection [5, 7]. Pooled analysis from different studies suggests a much higher sensitivity in the third week after symptom onset (ranging from 69.9 to 98.9%) compared with the first week (from 13.4 to 50.3%) [5]. Lijia et al. reported that antibodies against SARS-CoV-2 could be detected as early as 1.5–2 days, pointing towards the potential use of serological tests for early diagnosis [8].

However, the dynamics of immune response in SARS-CoV-2 remain ambiguous [9]. Furthermore, the use of the serological test as a stand-alone method for diagnosis is limited by several constraints, including false-positive results due to cross-reaction with other coronaviruses [10–12] or previous encounters with the virus [12]. The current study aimed to address these research gaps by exploring the sensitivity of SARS-CoV-2 IgG and IgM rapid tests in COVID-19 rRT-PCR-positive patients.

## Methodology

### Study Population and Sample Collection

The current study was approved by the Institutional Review Board (IRB) in Dr. Sulaiman Al Habib Medical Group. A consecutive cohort of 69 patients visited the Emergency Department of Al-Habib Hospital, Takhassusi branch in Riyadh, Saudi Arabia, for COVID-19-related symptoms or recent exposure to COVID-19–positive individuals and were enrolled from August 2020 to April 2021, after taking informed consent. Nasopharyngeal swabs for SARS-CoV-2 rRT-PCR analysis were obtained once at the beginning from all participants. Simultaneously, venous blood samples for COVID-19 IgG/IgM rapid tests were also collected (day 0 samples). Positive rRT-PCR cases were subjected to IgG/IgM rapid re-testing on days 7, 10 and 14.

The tests were immediately performed in reference laboratories. Registries containing the primary clinical data of patients, including the date of symptom onset, were created. Nasopharyngeal swab samples were directly analyzed for SARS-CoV-2 by rRT-PCR at Alhabib MD laboratory (Riyadh, Saudi Arabia), the reference laboratory for SARS-CoV-2 testing for all Alhabib Medical Group (HMG) branches. The blood samples were analyzed at the HMG Takhassusi laboratory. This study was performed in single center, and we faced difficulties in consenting patients. In addition, a large proportion of those who consented at the beginning for day 0 samples were lost during follow-up for subsequent samples. Moreover, the loss of these candidates in middle of the study led to a waste in the limited funding available and reduced our capacity to recruit more candidates. All of these factors reduced the sample size in this study.

### Real-Time PCR for Detection of SARS-CoV-2 RNA

According to the manufacturer's instructions, nasopharyngeal/oropharyngeal swabs were subjected to nucleic acid extraction with cell lysis buffer (Biospeedy). The presence of the *E*, *RdRP* and *N* genes of the SARS-CoV-2 virus were identified by rRT-PCR assay (Allplex 2019-nCoV Assay; Seegene). Samples were considered positive if all genes were detected, or if either, or both, of the *RdRP* and *N* genes were detected. If the *E* gene was detected alone, it was regarded as a presumptive positive for SARS CoV-2 and considered for repetition.

### Detection of IgG and IgM Antibodies Against SARS CoV-2

The Biozek COVID-19 IgG/IgM Rapid Test (Biozek Medical, Inzek B.V., Apeldoorn, Netherlands) is a qualitative membrane-based immunoassay for the detection of IgG and IgM antibodies against SARS CoV-2 in whole blood, plasma or serum samples. Venipuncture whole blood procedures were adopted and a dropper was used to transfer the whole blood to the specimen well. One full drop of blood was transferred to the well, then two drops of buffer were added. The results were read in 10 min. The results were interpreted in accordance with the manufacturer’s instructions. A colored line in the IgG section or/and IgM section indicated the detection of IgG or/and IgM in the presence of the control line. The Biozek COVID-19 Test was selected due to consistent results, the availability of supply and the affordability.

### Statistical Analysis

Frequency and percentage were used for descriptive analysis and cross-tabulation was used to identify the relationship between categorical variables. All data analysis was performed using SPSS version v23 (IBM Corp, Armonk NY, USA).

## Results

Of the 69 patients recruited, 36 (52.2%) were males, and 33 were females (47.8%). The mean age was 35.5 ± 11 years (range [r] 14–64 years). No significant relationship was found between age, sex, and positive COVID-19 cases by either chi-squared tests or Pearson’s correlation analysis. Overall, 45 (65.2%) patients tested positive by PCR. The rapid serological tests were not performed for 1, 3 and 9 patients on days 7, 10, and 14, respectively. The percentage of positive test results for IgG and IgM on days 0, 7, 10 and 14 are shown in Table 1. For IgG, the number of positive results were 3 (4.3%), 10 (14.7%), 20 (30.3%) and 26 (43.3%) on days 0, 7, 10 and 14, respectively. There was no positive result for IgM on day 0; however, there were 4 (5.9%), 7 (10.6%), and 8 (13.3%) positive IgM tests on days 7, 10, and 14, respectively. All positive results for IgM and IgG were also positive with the gold standard method, rRT-PCR.

**Table 1:** The proportions (%) of rapid IgM and IgG positive test results on days 0, 7, 10 and 14 out of the total number of samples (69)

	Day 0 *n* (%)	Day 7 *n* (%)	Day 10 *n* (%)	Day 14 *n* (%)
IgM positive	0 (0.0)	4 (5.9)	7 (10.6)	8 (13.3)
IgG positive	3 (4.3)	10 (14.7)	20 (30.3)	26 (43.3)

Table 2 summarizes the sensitivity of the IgG and IgM rapid test when compared to rRT-PCR. The IgG test exhibited sensitivity values of 6.7%, 22.7%, 47.6% and 72.2% on days 0, 7, 10 and 14, respectively. In comparison, the sensitivity values for the IgM test were 0%, 9.1%, 16.7% and 22.2%, respectively. This data clearly indicated the poor sensitivity of the IgM test in comparison to the IgG test.

**Table 2:** The sensitivities of the IgM and IgG assays compared with the gold standard method (PCR)

	PCR day 0		
	Positive	Negative	Measure
Day 0			
IgM positive	0	0	Sensitivity of IgM at day 0 = 0%
IgG positive	3	0	Sensitivity of IgG at day 0 = 6.7%
IgM negative	45	24	
IgG negative	42	24	
Day 7			
IgM positive	4	0	Sensitivity of IgM at day 7 = 9.1%
IgG positive	10	0	Sensitivity of IgG at day 7 = 22.7%
IgM negative	40	24	
IgG negative	34	24	
Day 10			
IgM positive	7	0	Sensitivity of IgM at day 10 = 16.7%
IgG positive	20	0	Sensitivity of IgG at day 10 = 47.6%
IgM negative	35	24	
IgG negative	22	24	
Day 14			
IgM positive	8	0	Sensitivity of IgM at day 14 = 22.2%
IgG positive	26	0	Sensitivity of IgG at day 14 = 72.2%
IgM negative	28	24	
IgG negative	10	24	

## Discussion

An initial study performed on the kinetics of antibody formation in 535 plasma samples from 173 COVID-19 subjects using the ELISA method found that the total seroconversion rate for IgG, IgM and total antibody was 64.7%, 82.7% and 93.1%, respectively, with a median seroconversion time of 14, 12 and 11 days, respectively. A cumulative seroconversion curve showed that the detection rate for both antibodies and IgM reached 100% at approximately 1 month after onset [13]. We found that the IgG-based assay sensitivity was initially low on day 0 and gradually increased on the subsequent days of testing. The sensitivity for IgM also increased with time but it was still very low with the highest sensitivity of 22.2% recorded on day 14. This finding is in line with the results of a previous systematic review of 57 studies that identified significant heterogeneity in the sensitivity of IgM and IgG antibody assays that gradually increased over time after the onset of symptoms [14].

Serological tests have emerged as a cost-effective and easy-to-use adjunct to rRT-PCR for diagnosing acute SARS-CoV-2 infection [5]. One major advantage of these serological tests is that they can identify individuals previously infected by SARS-CoV-2 who never underwent rRT-PCR testing during infection [5]. They can also estimate the time passed since infection, as IgM antibodies are indicators of recent infection, whereas the levels of IgG antibodies increase later [15–17].

In our study, the sensitivity of the IgG assay was much higher than for the IgM assay, thus, the Infectious Diseases Society of America (IDSA) recommended using the total antibody assay or assays designed for IgM in combination with IgG or IgG alone [18]. The reported data related to the sensitivity of serological assays varied. Pooled data from 38 studies with a total of 7848 individuals reported variable sensitivities from different assays, 90–94% from ELISA and CLIA-based methods followed by LFIA and FIA with sensitivities ranging from 80 to 89% [19]. Another meta-analysis determined the pooled sensitivity of various serological assays from 40 studies. The pooled sensitivity of ELISAs, CLIAs and LFIAs was 84.3% (95% CI 75.6–90.9%), 97.8% (46.2–100%) and 66% (49.3–79.3%), respectively [5]. This variation in sensitivity was possibly due to the timing of sample collection, the different products of the immunoassays used, variable immune response and serum immunoglobulin levels in the study subjects.

While serological assays offer a rapid approach for COVID-19 diagnosis [17, 20–22], the strength of antibody response varies based on several factors, including nutritional status, age, disease severity, and certain medications or infections [11, 13, 23]. Some studies have reported the presence of weak, delayed or an entirely absent antibody response in some of the individuals with positive rRT-PCR test results for SARS-CoV-2 [11, 13, 24]. Another limitation of serological assays is the delay in the production of antibodies, and thus, these tests give positive results much later than PCR [21, 23–25]. A third limitation of these assays is their cross-reactivity with other coronaviruses such as MERS-CoV, OC43 and HKU1 [26–28]. Also, a positive test result is not an assurance of complete immunity against COVID-19. Currently, the World Health Organization (WHO) does not recommend using serological assays to guide the decision-making process. However, these tests can be used for epidemiological surveys and to support vaccine development [29].

The results of our study combined with previously published reports indicate that these serological assays have low sensitivity compared to rRT-PCR based assays and cannot be used solely for the diagnosis of COVID-19. Combining the test results for rRT‐PCR and antibody detection can significantly improve the sensitivity of COVID‐19 diagnosis [17]. Furthermore, these serological assays can be used for epidemiological surveys and assist with vaccine development and improvement [29].

## Limitations of the Study

The limitations of our study include a relatively low number of patients due to funding issues and difficulties in obtaining patient consent. Secondly, only patients with positive results of PCR were included in the follow-up and further assessment of serological status. Thirdly, clinical evaluation of the study subjects was not performed.

## Conclusion and Future Prospects

Serological assays for the detection of infection with SARS-CoV-2 are rapid when compared to rRT-PCR. However, these assays yield lower sensitivity compared to standard rRT-PCR based assays. These tests also failed to detect the presence of the virus early in the course of infection, and a positive test result did not indicate that the patient was entirely immune to the virus. Nevertheless, these assays can be used for epidemiological and research purposes, to guide vaccine development, and to improve the sensitivity of COVID-19 diagnosis through standard rRT-PCR testing.

## Data Availability

Available on request from the corresponding author.

## Abbreviations

ACE2: Angiotensin-converting enzyme 2
CLIA: Chemiluminescence enzyme immunoassay
COVID-19: Coronavirus disease 2019
ELISA: Enzyme-linked immunosorbent assay
FIA: Fluorescence immunoassay
IDSA: Infectious Diseases Society of America
Ig: Immunoglobulin
LFIA: Lateral flow immunoassay
NAATs: Nucleic acid amplification tests
rRT-PCR: Real-time reverse transcription-polymerase chain reaction
SARS-CoV-2: Severe acute respiratory syndrome coronavirus 2
WHO: World Health Organization
